# Coil Embolization of Unruptured Distal Anterior Cerebral Artery Aneurysm Using a Marathon Microcatheter

**DOI:** 10.7759/cureus.24841

**Published:** 2022-05-09

**Authors:** Kenji Fukutome, Hiroyuki Ohnishi, Yoshihiro Kuga, Hideyuki Ohnishi

**Affiliations:** 1 Neurosurgery, Osaka Police Hospital, Osaka, JPN; 2 Neurosurgery, Ohnishi Neurological Center, Akashi, JPN

**Keywords:** marathon catheter, ed coil, distal anterior cerebral artery, coil embolization, aneurysm

## Abstract

Marathon is rarely used in coil embolization for an aneurysm; particularly, there have been no reports about distal anterior cerebral artery aneurysms. We have reported a case of successful use of Marathon in coil embolization in case of a distal anterior cerebral artery aneurysm. The patient was an 83-year-old woman. She had undergone coil embolization for an unruptured distal anterior cerebral artery aneurysm, which was discovered by chance. Our initial approach involved the use of a combination of Traxcess and Excelsior SL-10, but the use of SL-10 could not follow Traxcess because the right anterior cerebral artery from the right internal carotid artery had a sharp bifurcation. However, by switching to a combination of TENROU and Marathon, we could access the aneurysm. We thereby decided to continue the use of Marathon in order to complete the coil embolization. In coil embolization for an aneurysm, Marathon was found to be useful, depending on the location of the aneurysm and access route.

## Introduction

Marathon microcatheter (Medtronic, Minneapolis, Minnesota, USA) is a small-diameter flow-guided catheter that is sometimes used when using embolic material, peripheral lesions, or when approaching highly tortuous blood vessels, but it is rarely used for conventional coil embolization of aneurysms. Although there are restrictions on the coils that can be used, sufficient embolization of aneurysms is possible even with Marathon. To date, only nine cases have been reported in which Marathon was used for coil embolization of aneurysm, but no reports have been used in coil embolization of distal anterior cerebral artery (ACA) aneurysms [1-5]. We have reported a successful case using Marathon in coil embolization in case of a distal ACA aneurysm.

## Case presentation

**Figure 1:**
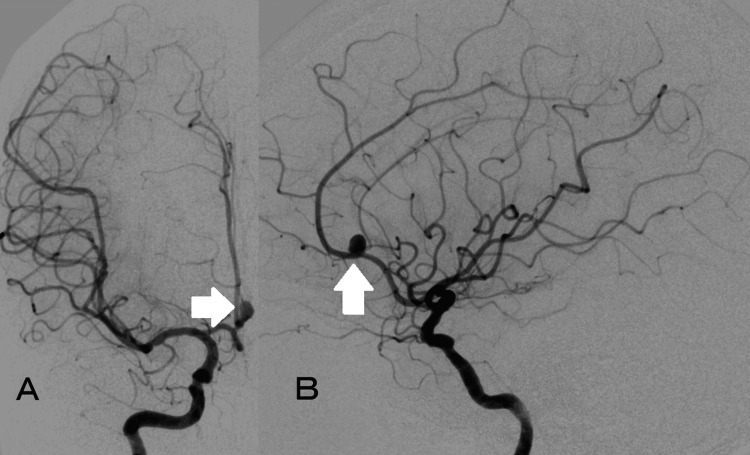
Angiogram of the right internal carotid artery The images showing a saccular aneurysm (white arrow) at the right anterior cerebral artery. (A: A–P view, B: lateral view)

The patient was an 83-year-old woman. She visited our hospital with a complaint of headache, and an unruptured distal ACA aneurysm was incidentally found by magnetic resonance imaging (MRI) examination. Cerebral angiography showed a saccular aneurysm with a maximum diameter of 4.9 mm, a height of 4.5 mm, and a neck of 2.3 mm at the bifurcation of the pericallosal-callosomarginal artery of the right ACA (Figures 1A-1B). Although she was elderly, she strongly hoped for its treatment, so we planned to perform coil embolization.

**Figure 2:**
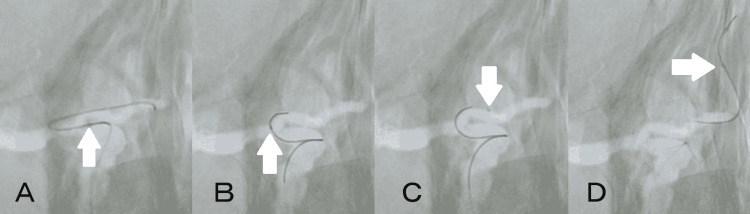
Approaching to the right ACA (A) Excelsior SL-10 could not follow the microwire, because the right ACA was sharply branched from the right ICA. (B–D) Marathon could enter the ACA even with little support from the microwire. White arrow indicates the tip of microcatheter. ACA: anterior cerebral artery; ICA: internal carotid artery

**Figure 3:**
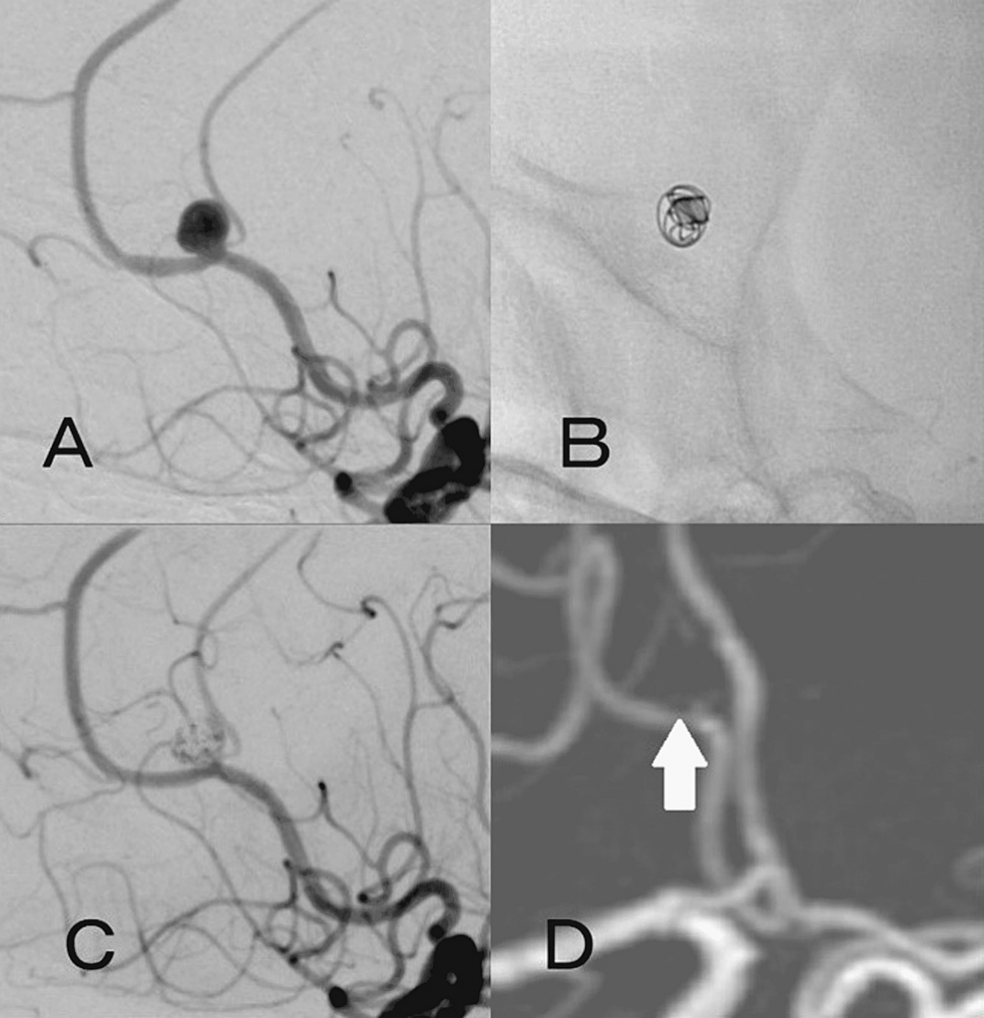
Intraoperative and postoperative images (A) Working angle of coil embolization. (B) The ED complex (3 mm × 6 cm) created a good framing by avoiding the origin of the pericallosal artery branching from the aneurysmal neck. (C) After inserting seven ED coils (27 cm of total length), only a slight depiction of the dome was noted, and the pericallosal artery was preserved. (D) No recurrence was observed in the magnetic resonance angiography a year after the surgery (white arrow). ED: electrodetachable

The operation was performed under general anesthesia. After placing a 4French (Fr) short sheath in the right femoral artery, it was replaced with 5Fr FUBUKI guiding sheath (Asahi Intecc, Seto, Aichi, Japan). After heparinization, FUBUKI was placed in the right internal carotid artery (ICA) using a 0.035-inch Radifocus guide wire (Terumo, Tokyo, Japan) and 4Fr OK2M 125 cm (Cathex, Nagoya, Aichi, Japan) as a coaxial system, and then 4Fr Cerulean G 115 cm (Medikit, Tokyo, Japan) as an intermediate catheter was placed in the ICA as far as possible. A 45° pre-shaped Excelsior SL-10 (Stryker, Kalamazoo, MI, USA) was attempted to enter using a Traxcess14 200 cm (Terumo, Tokyo, Japan), but the right ACA from the right ICA had a sharp bifurcation. Therefore, Traxcess could not reach A2, and when the tip of Traxcess was at A1 level, SL-10 bounced into the middle cerebral artery (MCA) together with Traxcess, making it impossible to enter the ACA (Figure 2A). Although the microguidewire was changed to CHIKAI black 18 (Asahi Intecc, Japan), SL-10 could not enter the ACA, so the microcatheter was changed to Marathon and the microguidewire was changed to 0.010-14 TENROU 200 cm (Kaneka Medics, Osaka, Japan). Like Traxcess, it was difficult to get TENROU into A2, but even if the tip of Traxcess was at A1 level, Marathon could follow and easily enter A1 (Figures 2B-2D). After that, we considered changing the microcatheter for embolization of aneurysm but decided to guide Marathon into the aneurysm with TENROU and embolize it with an electrodetachable (ED) coil (Kaneka Medics, Osaka, Japan). The ED complex 3 mm × 6 cm was inserted, and good framing was achieved, avoiding the origin of the pericallosal artery branching from the aneurysmal neck (Figure 3A). Subsequently, embolization was performed with ED extrasoft 3 mm × 4 cm, 2.5 mm × 4 cm × 2, 2.5 mm × 3 cm, and 2 mm × 3 cm. Although the aneurysmal dome was slightly visualized mainly on the dorsal side of the aneurysm by angiography, the embolization was completed because of the risk of obstruction of the pericallosal artery and the age of 83 years (Figure 3B). There was no particular problem with the operability of Marathon during embolization. Finally, angiography was performed to confirm that there were no obvious embolic complications, and treatment was finished. There were no complications after the operation, and no obvious recurrence was observed on the magnetic resonance angiography a year after the operation (Figure 3C).

## Discussion

Marathon is a flow-guided microcatheter with an outer diameter of 1.5 Fr and a flexible tip, often used to access small, tortuous vessels, especially used for the treatment of cerebral arteriovenous malformations and dural arteriovenous fistulas. The effective length is 165 cm, which is longer than the commonly used catheter for aneurysm embolization, so it is effective for access to distal lesions and tortuous blood vessels.

Only nine cases have been reported in which Marathon was used for coil embolization of aneurysms (Table 1) [1-5]. Becket et al. reported that Marathon was useful for distal posterior inferior cerebellar artery aneurysms because of its small diameter and long effective length [1]. Horie et al. used Marathon for distal anterior inferior cerebral artery aneurysm and distal MCA aneurysm, of which n-butyl-cyanoacrylate was used with coil for distal anterior inferior cerebral artery aneurysm [2]. They also described that Marathon, which could use various embolic substances, including Onyx (Medtronic, Minneapolis, Minnesota, USA), was useful. Stidd et al. reported that Marathon had a long effective length and was useful for two cases of anterior communicating artery aneurysm [4].

**Table 1:** Summary of the reported cases and the present case in which Marathon was used for embolization of aneurysms ACA: anterior cerebral artery; AcomA: anterior communicating artery; AICA: anterior inferior cerebellum artery; F: female; ICA: internal carotid artery; M: male; NBCA: n-butyl-cyanoacrylate; PcomA: posterior communicating artery; PED: pipeline embolization device; PICA: posterior inferior cerebellum artery

Case No.		Authors	Age/Sex	Location	Coil	Other material	Complication	Embolization
1	Stidd et al. [4]		80/M	AComA	Barricade	－	－	Good
2	Stidd et al. [4]		48/M	AComA	Barricade	－	Clot collecting within Marathon	Good
3	Stidd et al. [4]		48/F	ICA	Target ultrasoft	Neuroform EZ	－	Good
4	Horie et al. [2]		31/F	Distal AICA	ED infini, extrasoft ED extrasoft	NBCA	Facial palsy	Good
5	Horie et al. [2]		61/F	Distal AICA	ED extrasoft	－	－	Good
6	Beckett et al. [1]		52/F	PICA	Barricade	－	－	Good
7	Beckett et al. [1]		53/M	PICA	Barricade	－	－	Good
8	Uyama et al. [5]		66/F	ICA-PComA	ED extrasoft	Neuroform Atlas	－	Good
9	Sonobe et al. [3]		55/F	ICA	ED infini soft	PED	－	Good
10	Present case (2021)		83/F	Distal ACA	ED complex ED extrasoft	－	－	Good

They also reported that during stent-assisted coil embolization for an aneurysm in the tip of the ICA, the trans-cell technique with Marathon was easy because Marathon had a small diameter. Sonobe et al. reported that the small diameter of Marathon allowed the trans-cell technique to be used for ruptured case of intracavernous sinus carotid artery aneurysm after placement of a flow diverter [3]. Uyama et al. reported that during stent-assisted coil embolization of the ICA-posterior communicating artery aneurysm, the small diameter of Marathon made it easier to pass through a stented posterior communicating artery and perform the trans-cell technique [5]. In our case, ACA diverged from the ICA at an acute angle, but Marathon was useful because it was able to successfully enter A1. Other strategies are to replace the intermediate catheter with a more distally reachable one, such as Tactics (Technocrat, Kasugai, Aichi, Japan) in order to increase power of support, to place an occlusion balloon in order to prevent the microcatheter from bouncing into the MCA or to approach from the contralateral side via the anterior communicating artery. About the embolization of aneurysm, when TENROU reached the aneurysm, it might be considered to replace it with a commonly used microcatheter for embolization of aneurysm such as SL-10 using a long wire, but the procedure would be complicated and the system might slip down to the MCA. In our case, coil embolization was performed without replacing the Marathon, but the coil embolization was successful. Marathon was useful for access to A2/3 parts as in our case or more distal lesions, as well as tortuous vessels.

Regarding the treatment of distal ACA aneurysm, endovascular treatment was once reported to be difficult due to distal lesion, many bending points in which catheter control was difficult, relatively small aneurysm, and small diameter of parent artery [6-8]. However, recently, due to improvements in devices and techniques, the success rate of endovascular treatment has increased, and there are some reports that good results have been obtained [9-12]. A systematic review of endovascular treatment for distal anterior cerebral aneurysms, including ruptured cases, by Sturiale et al. reported a complete or near-complete embolization rate of 86% and a treatment failure rate of 8% [11]. One of the causes of unsuccessful treatment was that catheter control was difficult due to the distal lesion and many flexion points, and although not mentioned in detail, the bifurcation angle of the ACA from the ICA might also have an effect. There has been no report of coil embolization of distal ACA aneurysm using Marathon, but even in such cases with access route problems, it may be possible to solve it by an approach using Marathon as in our case.

On the other hand, Marathon also has disadvantages. One is that the inner lumen at the tip is as small as 0.013 inch, and the coils that can be used are limited. In previous reports, ED extrasoft, ED infini extrasoft, Barricade (Blockade Medical, Irvine, CA, USA), and Target ultrasoft (Stryker Neurovascular, Fremont, CA, USA) were used for embolization of aneurysms, and good embolization was obtained in all cases (Table 1) [1-5]. However, due to its long effective length of 165 cm, the Y adapter must be temporarily removed when using the Barricade or Target, and irrigation in the Marathon will stop [1,2]. Fortunately, there was no effect on the patient, but in previous reports, only one case was reported to have a thrombus in Marathon due to the effect of temporarily stopping irrigation when using Barricade [4]. As for the ED coil, the pusher wire is as long as 187 cm, so there is no need to remove the Y adapter.

Another disadvantage is that Marathon does not have the second marker. It becomes difficult to determine the detachment point of the coil when the tip becomes invisible, such as when the tip of Marathon is in the coil mass in the latter half of the embolization. At that time, if the pusher wire is inserted deeply, there is a risk of perforation, and if the insertion is shallow, the coil may deviate to the parent artery. However, for the ED coil, the detachment point can be recognized by an alarm even if the tip of the microcatheter cannot be seen. Therefore, when performing coil embolization using Marathon, the ED coil is considered to be the safest coil. In our case as well, the ED coil was very useful because the tip of the Marathon gradually became invisible due to the coil mass. Regarding the ED coil, a new 3D-shaped i-ED coil (Kaneka Medics, Osaka, Japan) has recently appeared, and it has become possible to deal with various aneurysms, so it is expected that the method used in our case will be useful in more cases.

## Conclusions

We reported a case in which coil embolization was successfully performed using Marathon for an unruptured distal ACA aneurysm. In coil embolization for an aneurysm, Marathon was found to be useful, depending on the location of the aneurysm (for example, distal lesion) and access route (for example, tortuous or small vessels).
